# Gamabufotalin Suppresses Colorectal Cancer Growth via Oxidative Stress-Induced Apoptosis and DNA Synthesis Inhibition

**DOI:** 10.5812/ijpr-169859

**Published:** 2026-04-14

**Authors:** Hui Nie, Anfang Cui, Zhicheng Sun, Zhaoyang Li, Jianhua Guo, Shuhao Fan, Xiangchen Wang, Yong Xia

**Affiliations:** 1 School of Pharmacy, Shandong First Medical University (Shandong Academy of Medical Sciences), Jinan, China; 2 College of Basic Medicine, Jining Medical University, Jining, China; 3 Key Laboratory for Chronic Non-communicable Diseases, Institute of Precision Medicine, College of Medical Engineering, Jining Medical University, Jining, China

**Keywords:** Gamabufotalin, Colorectal Cancer, Oxidative Stress, Apoptosis, DNA Synthesis

## Abstract

**Background:**

Colorectal cancer (CRC) continues to be a major cause of cancer-related mortality worldwide, creating an urgent need to develop novel therapeutic agents that are both highly effective and minimally toxic. Gamabufotalin (GA), a bioactive bufadienolide compound, has shown promising antitumor potential, including against CRC; however, its precise mechanisms of action in CRC remain incompletely understood.

**Objectives:**

The aim of this study was to investigate the therapeutic effect of GA on CRC and explore the underlying molecular mechanisms.

**Methods:**

A combination of in vitro and in vivo approaches was employed, including cell viability assays, colony formation, EdU incorporation, cell cycle and apoptosis analyses by flow cytometry, ROS and mitochondrial membrane potential detection, as well as integrated transcriptomic and proteomic profiling. The in vivo antitumor efficacy was further validated in a nude mouse xenograft model.

**Results:**

Gamabufotalin exhibited potent, dose-dependent anti-CRC activity in vitro (IC_50_: 24-30 nM) with high selectivity over normal cells. It triggered mitochondrial apoptosis via ROS generation and arrested the cell cycle, suppressing DNA synthesis. Integrated omics revealed TP53I3/PIG3 upregulation and RFC3/NUCKS1 downregulation as key mechanisms. Critically, these effects converged to suppress tumor growth in vivo without systemic toxicity.

**Conclusions:**

Gamabufotalin selectively and potently inhibits CRC through dual mechanisms: Inducing ROS-mediated apoptosis and suppressing proliferation. With a favorable therapeutic index and defined molecular targets, GA represents a promising candidate for CRC therapy.

## 1. Background

Colorectal cancer (CRC) is the third most common cancer and the second leading cause of cancer-related death worldwide. The increase in its incidence is closely related to the modern lifestyle characterized by reduced physical activity and a high-fat diet. Current treatments, such as radiotherapy and targeted therapy, are often limited by toxicity and cost; thus, there is an urgent need for more cost-effective and efficient treatment (1-4). Therefore, it is imperative to investigate novel therapeutic methods and drugs aimed at improving outcomes for individuals diagnosed with CRC. Natural products are promising sources for anticancer drugs, offering diverse biological activities and a favorable safety profile (3). Compounds such as apigenin and resveratrol have shown antitumor potential and, when combined with chemotherapy, may improve outcomes by enhancing efficacy, reducing drug requirements, overcoming resistance, and reducing side effects. Therefore, identifying low-toxicity, high-efficacy natural compounds remains a key focus in current cancer research (2, 5, 6). HuaChanSu (HCS) is a purified component extracted from the skin of the Chinese toad. Its anti-cancer effect is derived from the bufalin substances in HCS, and its clinical effect is significant. It is extensively utilized in the treatment of various advanced malignancies, including gastric cancer, CRC, bladder cancer, and hepatocellular carcinoma (7, 8). Gamabufotalin (GA), a key bioactive monomer isolated from toad-derived Traditional Chinese Medicine, has demonstrated therapeutic potential against several types of cancers such as lung cancer, glioblastoma, and liver cancer (9-11). With minimal toxic side effects and proven potent anti-cancer activity, GA represents a promising candidate for further investigation (7). However, the impact of GA on CRC and its underlying mechanisms remain incompletely understood.

## 2. Objectives

Objectives This study investigated the anti-cancer mechanism of GA in CRC. These results may provide valuable ideas and strategies for the treatment of CRC, and help to develop novel anti-CRC therapeutic drugs based on GA.

## 3. Methods

### 3.1. Drugs and Reagents

Gamabufotalin, sourced from TargetMol, is derived from bufotenine and dissolved in dimethyl sulfoxide (DMSO) as the solvent to prepare a 10 mM stock solution stored at -20°C; after serial dilution to the experimental GA concentrations (0 - 100 nM), the final concentration of DMSO in the cell culture medium was 0.01% (v/v), which was verified to have no cytotoxicity on the tested cells in preliminary experiments. Phosphate-buffered saline (PBS), dimethyl sulfoxide (DMSO), and neutral formalin were all procured from Solarbio Technology Ltd.

### 3.2. Cell Culture

SW480, HCT-116, NCM460, and 293T human colon cancer cell lines were procured from the American Type Culture Collection (ATCC). DMEM medium (06-1055-57-1ACS, BI, Israel) was used to cultivate the cells, supplemented with 10% fetal bovine serum (FBS, A3160801, Hyclone, USA) and 1% penicillin-streptomycin (P/S, UB89609, GBICO, USA). The cultures were kept in a 37°C incubator with an atmosphere of 5% CO_2_.

### 3.3. Cell Viability Assay

Once the 96-well plates had been seeded with cells at a density of 25% and the density had risen to around 40%, they were then exposed to GA (0 - 100 nM) for 48 hours of treatment. Subsequently, following the instructions provided by the CCK-8 kit (CK04, Japan), the relative cell activities of SW480, HCT116, HT-29, NCM460, and 293T after GA treatment were evaluated. All the experiments were repeated at least three times independently.

### 3.4. Cell Morphology Assay

SW480 cells were plated in 12-well plates at a 25% confluency. After they attached, their starting shape was recorded (T0). They were then treated with varying concentrations of GA (0 - 100 nM) for 48 hours, and changes in cell appearance throughout the experiment were photographed using an inverted microscope (Nikon, Japan).

### 3.5. Cell Migration Experiments

In accordance with the manufacturer's instructions, the insert (80469, IBIDI, Germany) was affixed to the bottom of a 12-well plate. Subsequently, SW480 cells were seeded into the same plate. The insert was removed once the cells reached approximately 80% confluence. A scratch measuring 500 µm in width was then created. Gamabufotalin (0 - 100 nM) was added for treatment and incubated for a duration of 48 hours. The width of the cell scratch was observed and documented using a microscope (Nikon, Japan). Three independent experiments were conducted, and in each experiment, three separate culture wells were used.

### 3.6. Cell Membrane Staining Assay

SW480 cells at a density of 30% were seeded in 12-well plates and incubated with GA (0 - 100 nM) for 24 hours before being washed with PBS and fixed with 4% paraformaldehyde for 15 min. Subsequently, after three washes with PBS, the cells were stained with a double dye solution of Hoechst 33342 (diluted at 1:1000, C1022, Beyotime, China) and Dio (diluted at 1:1000, C1038, Beyotime, China) for a duration of 20 minutes. Images were subsequently taken using a Nikon, Japan fluorescence inverted microscope.

### 3.7. Clone Formation Experiments

Twelve-well plates were used to seed SW480 cells at an initial confluency of 30%, which were then incubated at 37°C in a 5% carbon dioxide environment. Once colony formation was observed, varying concentrations of GA were introduced, and the cultures were maintained for a duration of nine days, with medium changes occurring every three days. After drug treatment, 4% paraformaldehyde was used to fix the cells at room temperature for fifteen minutes, followed by staining with KGA229 (KeyGEN BioTECH, China) crystal violet staining solution for five to ten minutes. The colonies were then documented under an inverted microscope (Nikon, Japan).

### 3.8. Live/Dead Cell Detection

Twelve-well plates were utilized to cultivate SW480 cells, which were then subjected to a range of GA concentrations (0 - 100 nM) at an estimated cell density of 40% for 36 hours. Cell viability was evaluated using the Calcein AM Cell Viability Assay Kit (C2013FT, Beyotime, China), followed by microscopic examination of the stained cells (Nikon, Japan).

### 3.9. Measurement of DNA Synthesis Rate by EdU Method

SW480 cells were seeded in 12-well plates at a density of 30% until the density reached about 50%, and then the cells were treated with GA (0 - 100 nM) for 24 hours. The rate of DNA synthesis in SW480 cells treated with GA (0 - 100 nM) was assessed using the EdU-594 cell proliferation assay reagent (C0078S, Beyotime, China). Staining results were documented under a microscope (Nikon, Japan) and subsequently quantified using ImageJ software. All assays were performed with the use of three biological replicates and three technical replicates within each group.

### 3.10. Cell Cycle Assay

SW480 cells treated with GA (0 - 100 nM) for 24 hours were harvested by centrifugation. After 70% cold ethanol was added, the samples were incubated overnight at 4°C. Centrifugation at 300 xg then caused the supernatant to be discarded, and the cells were resuspended in a solution of propidium iodide (PI) (KGA214, KeGEN BioTECH, China), 0.2% Triton X-100 (P0096, Beyotime, China), RNase (ST579, Beyotime, China), and PBS. Subsequently, further analysis was conducted using a flow cytometer (Beckman Coulter, CytoFLEX, USA). The experiment was performed with three independent biological replicates.

### 3.11. Detection of ROS by H2DCFDA

At 37°C, SW480 cells treated with GA were incubated in a carbon dioxide incubator for 30 minutes with H2DCFDA. To evaluate the alterations in ROS levels induced by the cell-permeable ROS probe H2DCFDA (HY-D0940, USA), Beckman flow cytometry (Cytomics FC500, USA) was employed to measure intracellular ROS levels. To determine whether ROS contributed to the observed inhibition of cell activity, SW480 cells were pretreated with the antioxidant N-acetylcysteine (NAC) (ST1546, Beyotime, China) for one hour and subsequently exposed to 50μM NAC for an additional 48 hours. Finally, cell viability was evaluated using the CCK-8 assay kit.

### 3.12. Mitochondrial Membrane Potential Staining Experiment

After treating SW480 cells with different concentrations of GA for 24 hours, the JC-1 mitochondrial membrane potential reagent mixture was prepared according to the instructions of the JC-1 reagent kit. The cells were incubated in the dark for 25 minutes, then washed with PBS. Afterwards, Hoechst 33342 (C1022, Beyotime, China) was added to stain the cell nuclei for 10 minutes. The procedure was carried out using a fluorescence microscope (Nikon, Japan). The experiment was performed with three independent biological replicates.

### 3.13. Apoptosis Detection

SW480 cells treated with GA were digested using EDTA-free trypsin (T1350, Solarbio, China). After digestion, the cells were incubated in the dark for 20 minutes, having been mixed with Annexin V-FITC (C1062M, Beyotime, China) and PI (KGA214, KeyGEN BioTECH, China). The apoptosis rate was subsequently assessed and recorded via flow cytometry (Beckman, Cytolfex, USA). The experiment was repeated independently three times, with each biological replicate consisting of three technical replicates.

### 3.14. Immunofluorescence Staining

After 24 hours of GA treatment, SW480 cells were fixed in 4% paraformaldehyde (PFA) for 15 minutes. Subsequently, permeabilization was applied with 0.3% Triton X-100 for an additional 15 minutes, followed by blocking with 3% BSA for 30 minutes. The Ki67 antibody (1:3000, ab15580, Abcam, UK) was incubated overnight at 4°C. Finally, the cells were exposed to Coralite 488-labeled pure goat anti-rabbit IgG (H+L) (SA00013-2, Proteintech, China) for a period of 1.5 hours. After staining the cytoskeleton with Red594 F-actin tracker (C2205S; Beyotime, China) and incubating with H33342 for an extra 10 minutes, the cells were then washed with PBS and viewed under a fluorescence inverted microscope (Nikon, Japan).

### 3.15. Xenograft in Nude Mice

The BALB/c-nude mice utilized in this study were procured from Jinan Pengyue Laboratory Animal Technology Co., LTD. For the experiment, a total of 10 female mice, each weighing approximately 20 g, aged 6 weeks, were chosen. Female BALB/c-nude mice were injected subcutaneously with CRC SW480 cells. Upon reaching 100 mm³ of tumor volume, the mice were randomly divided into two groups (each containing five nude mice). The GA treatment group received an injection of 0.058 mg/kg every three days, while the control group was given DMSO at the same volume and frequency. Mice were euthanized by carbon dioxide inhalation after 10 injections; their CRC xenografts were then harvested, weighed, and photographed.

### 3.16. H&E Staining

Using a rotary paraffin microtome (LEICA RM2235), 4.0 um thick tissue samples were sliced from pre-prepared paraffin sections. Subsequently, the sections were stained with a G1120 staining kit (Chabi, China) and then imaged using a pathological section scanner (3DHISTECH, Hungary).

### 3.17. Immunohistochemical Staining

After antigen retrieval, the tissue sections were subjected to boiling with a modified sodium citrate repair solution (072420210224, Bayes, China) for treatment. Following this step, the cells were blocked using goat serum (17H18B09, Bost, China) for 30 minutes before incubation with the primary antibody Ki67(ab15580, Abcam, USA) at a dilution of 1:350 in 1% BSA. Staining was performed using a DAB kit (17I07A22; Bost, China), and the results were documented accordingly. Finally, dehydration was carried out, and the slides were mounted. Scanning observations were conducted using a panoramic stage (3DHISTECH, Hungary).

### 3.18. Western Blotting

Protein extraction was performed using GA-treated SW480 cells, and the reagent used was RIPA lysis buffer (P0013B, Beyotime, China). Electrophoresis of the protein extracts on SDS polyacrylamide gels was conducted, and they were then transferred to PVDF membranes for immunoblotting. The primary antibodies used in this study were TP53I3/PIG3 (1:1000,14828-1-AP, Proteintech, China), β-Actin (1:2000, GB11001, Servicebio, Wuhan, China), and Cleaved caspase-3 (1:500; WL0217; Wanlei, China). Following incubation with the primary antibodies, HRP-labeled secondary antibodies were utilized to detect the immune response signals. The secondary antibody used was HRP-conjugated sheep anti-rabbit IgG (1:3000; AS014; AB-clonal; Wuhan, China), which was then developed with an ECL hypersensitive detection reagent (P0018AS; Beijing, China).

### 3.19. Molecular Docking

The interaction proteins of GA in CRC cells were investigated using the Protein Data Bank (PDB). The database code for the MDM2 protein is 4hbm. Molecular docking simulations were performed using AutoDock software. For subsequent docking procedures, three rotatable bonds of GA were activated, and a grid box was configured with dimensions of 40 × 40 × 40Å³. To account for the flexibility of the protein, a soft docking feature was employed. The results from the protein-ligand docking were evaluated based on binding affinity (kcal/mol). All structural images were generated using PyMOL software (version 2.0, Schrodinger, LLC).

### 3.20. Proteomics

To evaluate the effects of GA on protein expression levels, protein extraction was performed on three control samples (CTL1, CTL2, CTL3) and three samples treated with 25 nM GA for a duration of 24 hours (GAT1, GAT2, GAT3). The proteomic analysis utilized the SDT method and was conducted in Shanghai, China. Each sample consisted of a total cell count of 1.5 × 10^7^ cells. Subsequent proteomic analyses and data conversion tasks were carried out by a specialized protein technology company based in Shanghai, China.

### 3.21. Transcriptome Analysis

For transcriptome sequencing, three GA-treated samples and three control samples were lysed using Trizol reagent, respectively. Library construction, mRNA sequencing, and bioinformatics analysis were performed at Biomarker (Beijing, China).

### 3.22. Statistics

Statistical analyses were performed using GraphPad Prism software. Normality and homogeneity of variance were examined prior to statistical testing. All the experiments were conducted with three repetitions. For comparisons between two independent groups, an unpaired Student’s *t*-test was used. For comparisons among three or more groups, one-way ANOVA was applied. A P-value < 0.05 was considered statistically significant.

## 4. Results

### 4.1. Gamabufotalin

**Figure 1.**
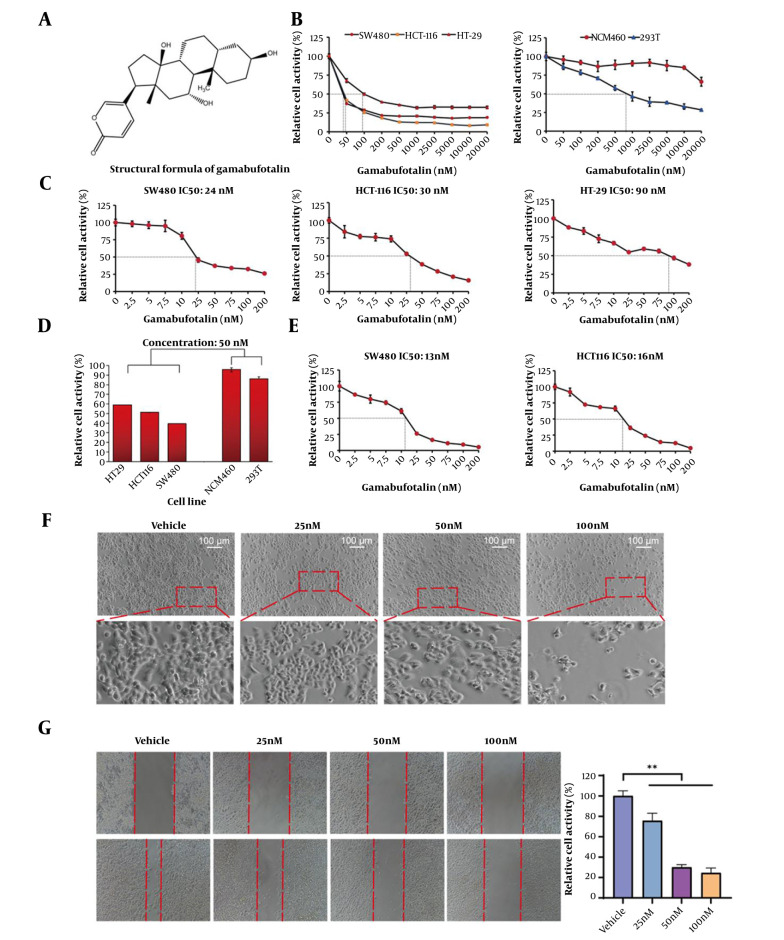
In vitro experiments demonstrated that gamabufotalin (GA) has a dose-dependent inhibitory effect on colorectal cancer (CRC) cells. A, the chemical formula of GA; B, SW480, HCT-116, HT-29, NCM460, and 293T cells were treated with 0-20000 nM GA for 48 hours, and viability was initially determined using the CCK-8 kit. Each concentration point was repeated six times (n = 6). C, CRC SW480, HCT-116, and HT-29 cells were co-incubated with GA at concentrations ranging from 0 to 200 nM for 48 hours, and the relative cell viability of each experimental group was evaluated using the CCK-8 kit. D, the cell viability of SW480, HCT-116, HT-29, NCM460, and 293T cells was measured using the CCK-8 kit at a GA concentration of 50 nM. E, the cell viability of CRC SW480 and HCT-116 cells was measured under a serum concentration of 1% in the culture condition. F, SW480 cells were treated with GA for 48 hours, and the morphological changes in SW480 cells caused by GA were photographed using an inverted microscope. G, SW480 cells were treated with different concentrations of GA for 24 hours, and the width of the scratch was measured, and the cell scratch closure rate was calculated. Post hoc analysis using LSD was performed after one-way ANOVA to evaluate significance, ** P < 0.01.

Exhibited a Dose-Dependent Inhibitory Effect on the Proliferation of CRC Cells The chemical structure of GA was shown in Figure 1A. CCK-8 assay results (Figure 1B–D) revealed that GA significantly inhibited the proliferation of CRC cells in a dose-dependent manner. The IC_50 _values of GA in SW480, HCT-116, and HT-29 cells were 24 nM, 30 nM, and 90 nM, respectively. Notably, GA demonstrated relatively low cytotoxicity toward normal human colonic mucosal epithelial cells (NCM460) and renal cells. Furthermore, as demonstrated in Figure 1E, the viability of SW480 and HCT-116 CRC cells decreased under 1% serum culture conditions. Gamabufotalin treatment induced significant morphological changes in SW480 cells, including increased cellular folding and elongation at higher drug concentrations (Figure 1F). Additionally, Figure 1G showed that GA suppressed the migratory capacity of SW480 cells in a dose-dependent manner, with cell motility progressively declining as GA concentrations increased.

### 4.2. Gamabufotalin Significantly Inhibited the Proliferation of Colorectal Cancer Cells and Triggered Their Death

**Figure 2.**
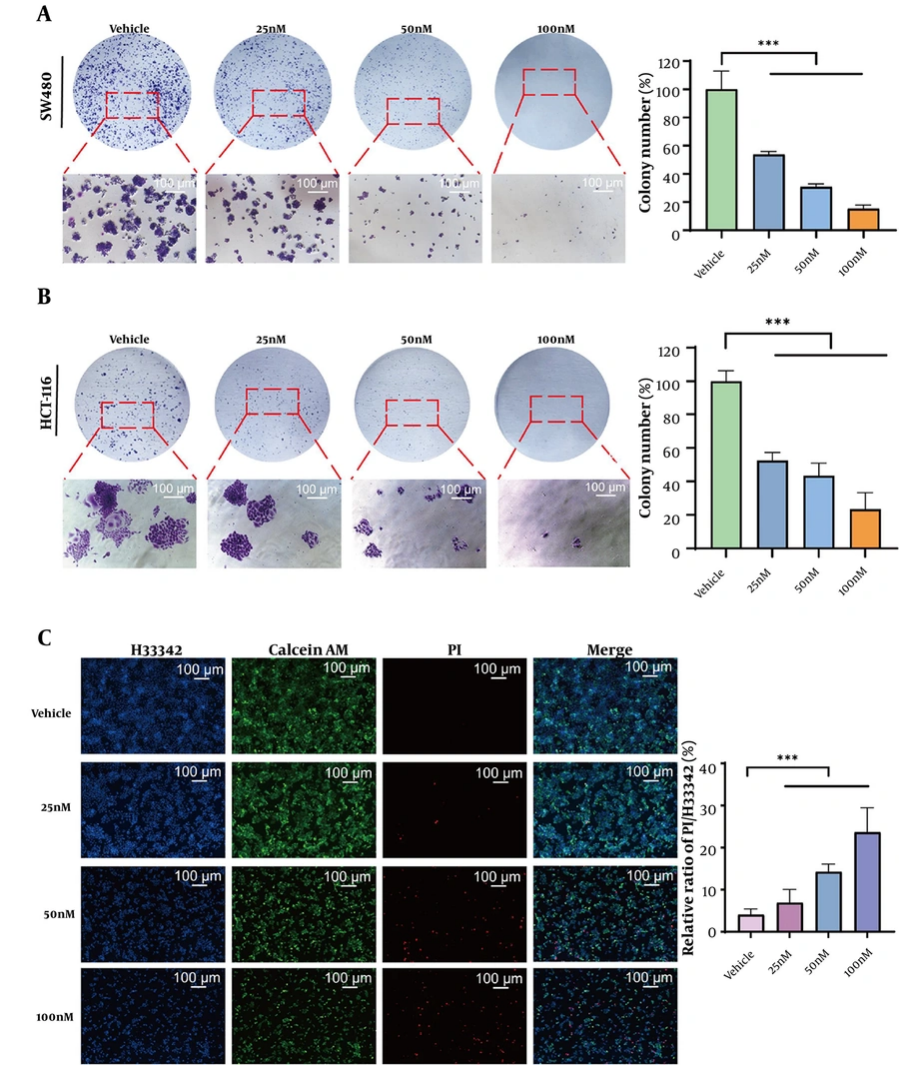
Gamabufotalin (GA) inhibits the proliferation of colorectal cancer (CRC) cells and triggers their death. A and B, SW480 and HCT-116 cells were treated with different concentrations of GA for 12 days, and the cell colonies stained with crystal violet were observed using an inverted microscope. Significance was evaluated using one-way ANOVA and subsequent LSD post hoc analysis, *** P < 0.001. C, SW480 cells treated with GA were stained with Hoechst 33342, Calcein AM, and PI, and the staining results were recorded using a fluorescence inverted microscope (Hoechst 33342: Blue, Calcein AM: Green, PI: Red).

During tumorigenesis, cellular proliferation and apoptosis served as two fundamental regulatory mechanisms. Our experimental findings established that GA potently inhibited the clonogenic potential of both SW480 and HCT-116 cell lines (Figure 2A and B). Figure 2C demonstrated that live/dead cell staining revealed a concentration-dependent decrease in viable cells and a concomitant increase in dead cells following GA treatment.

### 4.3. Gamabufotalin Significantly Suppressed the DNA Synthesis Rate in CRC Cells

**Figure 3.**
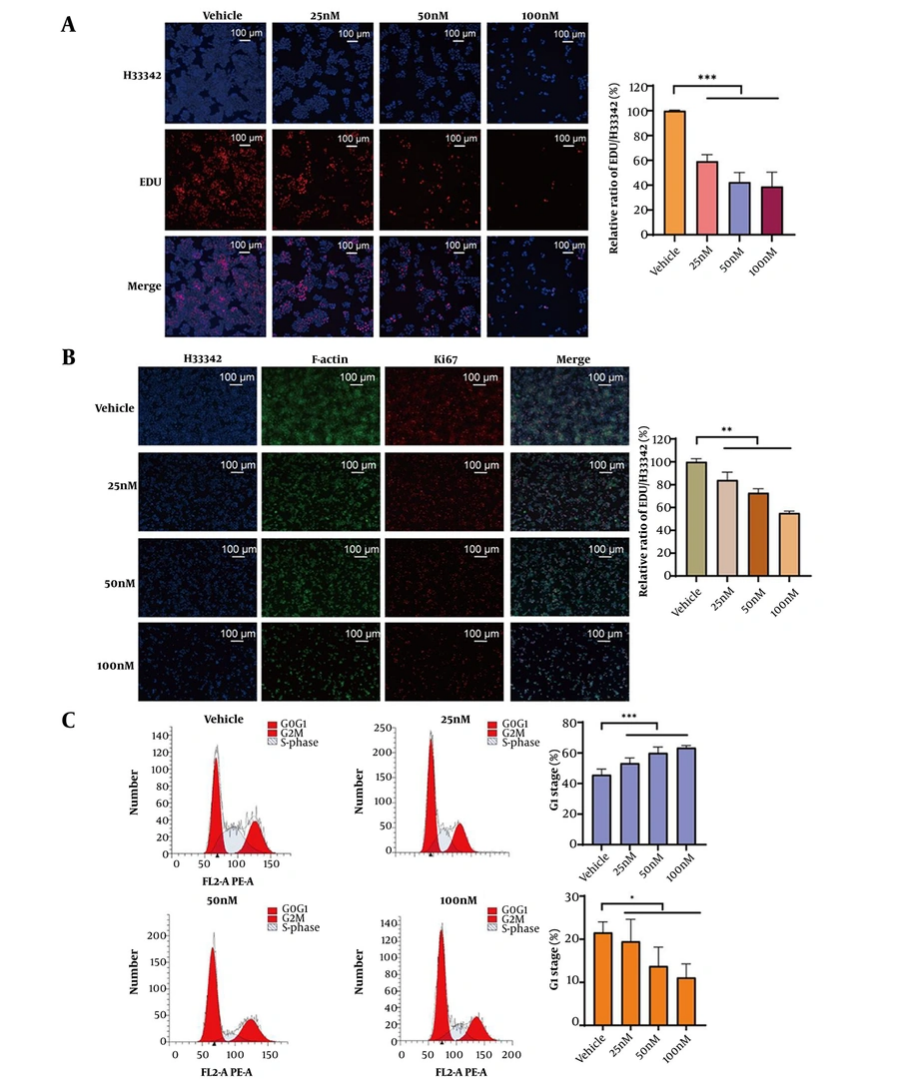
Gamabufotalin (GA) inhibits the proliferation of colorectal cancer (CRC) cells by inhibiting DNA synthesis. A, SW480 cells were treated with different concentrations of GA and stained with EdU and Hoechst 33342, and cell proliferation was observed using an inverted fluorescence microscope (EdU: Red, Hoechst 33342: Blue). *** P < 0.001. B, the proliferation of cells expressing Ki67 protein was detected using cell immunofluorescence. Hoechst 33342 was blue fluorescence, Ki67 was red fluorescence, and F-actin was green fluorescence. Significance was evaluated using one-way ANOVA and subsequent LSD post hoc analysis, ** P < 0.01. C, SW480 cells treated with GA were stained with PI, and the cell cycle of stained cells was detected using flow cytometry (* P < 0.05).

The DNA synthesis rate served as a key parameter for evaluating cell proliferation, activity, and physiological state. In this study, the EdU assay demonstrated that GA significantly inhibited DNA synthesis in SW480 cells (Figure 3A). Following GA treatment, cell numbers progressively declined. As GA concentrations increased, the relative EdU/Hoechst 33342 ratio exhibited a gradual reduction, demonstrating GA's effective suppression of DNA replication in SW480 cells. Furthermore, as a canonical proliferation marker, Ki67 mediates critical mitotic functions. Quantitative immunofluorescence analysis (Figure 3B) demonstrated a concentration-dependent attenuation of Ki67 signal intensity upon GA exposure, corroborating its anti-proliferative efficacy. Flow cytometric analysis revealed that GA treatment altered the cell cycle distribution of SW480 cells, with particularly pronounced effects on S-phase progression. Increasing GA concentrations led to a progressive decrease in the S-phase cell population (Figure 3C). These collective findings established that GA inhibited DNA synthesis through multiple mechanisms.

### 4.4. The Intracellular ROS Levels Following Gamabufotalin Treatment Were Quantified Using the H2DCFDA Fluorescent Probe

**Figure 4.**
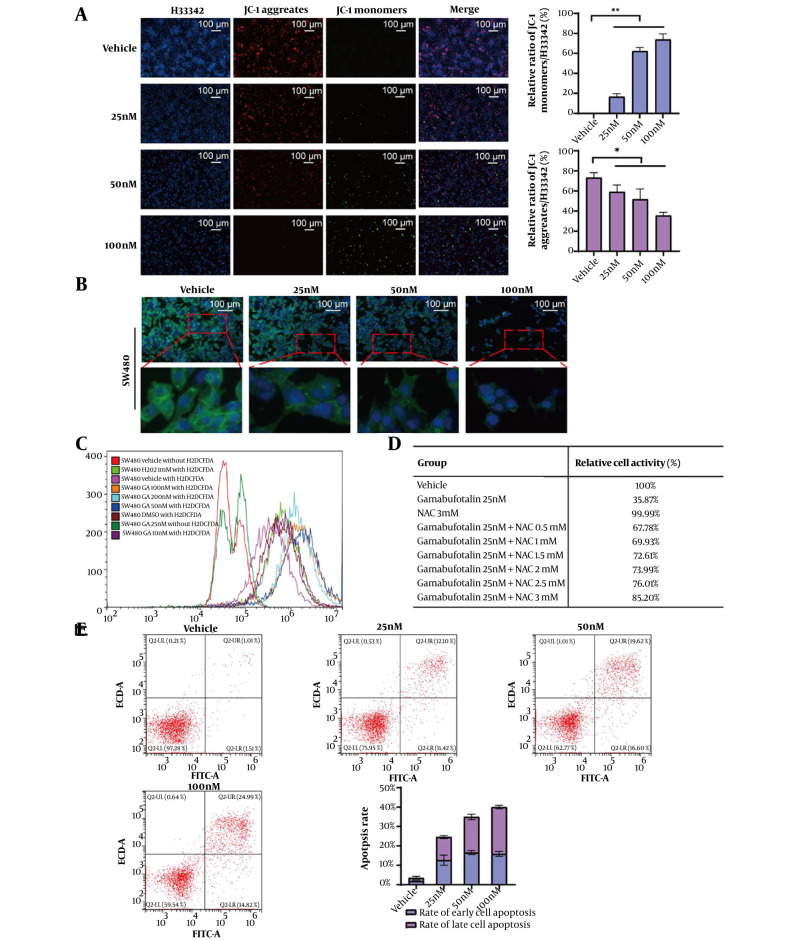
Gamabufotalin (GA) induces oxidative stress and promotes cell apoptosis. A, the changes in mitochondrial membrane potential in SW480 cells were detected using a mitochondrial membrane potential detection kit. Significance was evaluated by one-way ANOVA followed by LSD post-hoc analysis, * P < 0.05, ** P < 0.01. B, SW480 cells treated with GA were stained with Dio and Hoechst 33342, and the cell membrane showed green fluorescence and the cell nucleus showed blue fluorescence. C, the ROS level in SW480 cells was detected using the H2DCFDA fluorescence probe by flow cytometry. D, NAC antioxidants rescued the SW480 cells treated with GA, and the relative cell viability was detected by the CCK-8 kit. E, the apoptosis of stained cells was detected by flow cytometry. The apoptosis rate was determined by flow cytometry. The X-axis represents the FITC intensity of Annexin V on the outer membrane, and the Y-axis represents the PI staining intensity. The Q2-LR quadrant corresponds to the population of early apoptotic cells, and the Q2-UR quadrant corresponds to the population of late apoptotic cells.

The results of mitochondrial membrane potential detection showed that the membrane potential of SW480 cells treated with GA was significantly reduced (Figure 4A). Additionally, higher concentrations of GA led to the gradual death of SW480 cells, accompanied by severe damage to the cell membranes (Figure 4B). As illustrated in Figure 4C, GA triggered oxidative stress, with intracellular ROS levels escalating dose-dependently. Pretreatment with 3 mM NAC restored relative cell viability to ~85.2% (Figure 4D), confirming GA-induced oxidative stress-mediated cell death. To further investigate, flow cytometric apoptosis analysis was conducted. The results established that GA promoted apoptotic cell death in a concentration-dependent manner, reaching a 38% apoptosis rate at 100 nM GA (Figure 4E). 

### 4.5. To Investigate the Anticancer Mechanisms of Gamabufotalin in Colorectal Cancer, We Performed Proteomic and Transcriptomic Sequencing Analyses

**Figure 5.**
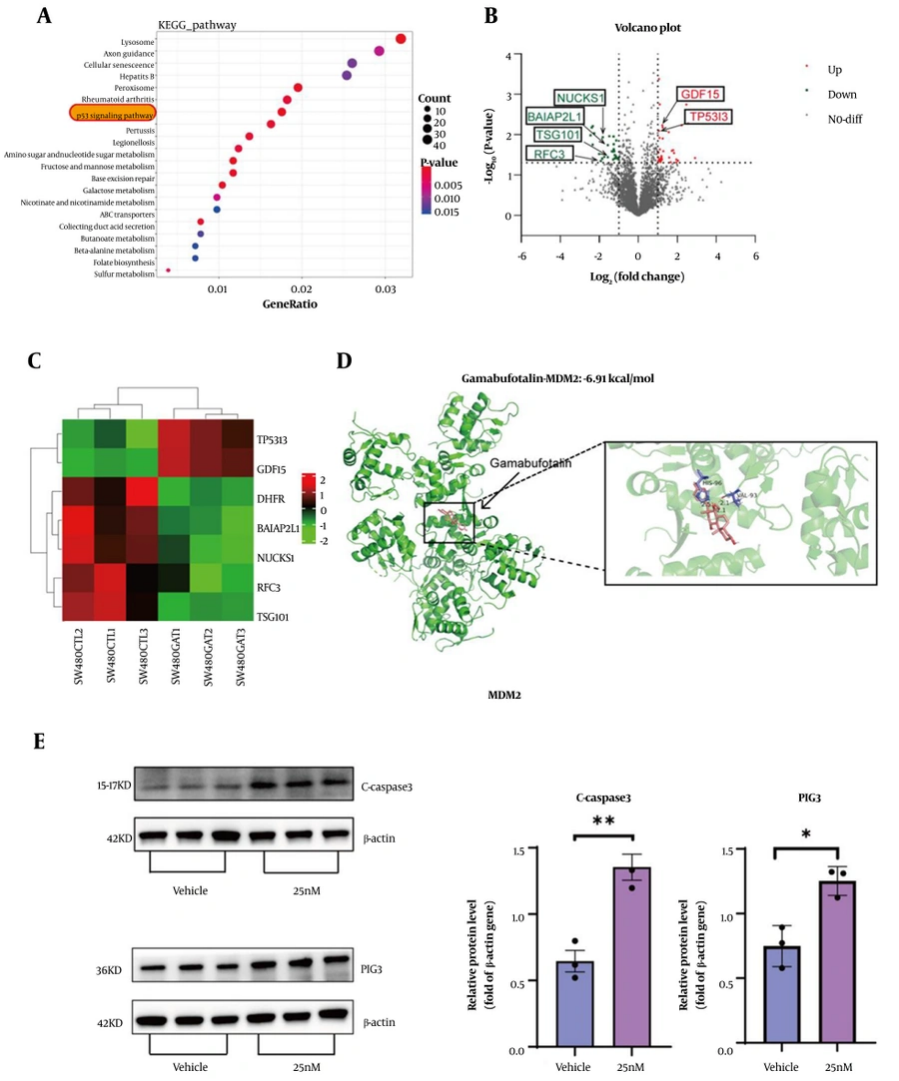
Gamabufotalin (GA) prevents colorectal cancer (CRC) by inducing cell apoptosis. A, KEGG enrichment analysis of transcriptomics showed the top 20 KEGG pathways after GA treatment. Through KEGG enrichment analysis, the P53-related pathway ranked high in both enrichment factors and significance (the related pathways are marked with red circles). B, the protein volcano plot shows the changes in protein levels after GA treatment. Red indicates increase, while green indicates decrease. C, the proteomics heatmap shows that GA upregulated the protein expression related to oxidative stress and apoptosis, such as TP53I3/PIG3 and GDF15, and downregulated the protein expression related to cell proliferation, such as RFC3, NUCKS1, TSG101, and BAIAP2L1. D, the docking structure of GA and the MDM2 molecule. GA and MDM2 are stained green and pink, respectively. E, the protein expression levels of β-actin, PIG3, and Cleaved Caspase-3 were determined by Western blot. Quantification was performed using the peak area of the relative protein expression gray value. Analysis was conducted using a two-sided *t*-test, ** P < 0.01, * P < 0.05.

Figure 5A To investigate the anticancer mechanism of GA, we performed integrated proteomic and transcriptomic sequencing followed by KEGG pathway analysis, which identified significant enrichment of the p53 signaling pathway. Figure 5B displays the volcano plot of differentially expressed genes (DEGs) comparing GA-treated and control groups. Gamabufotalin treatment significantly downregulated RFC3, NUCKS1, TSG101, and BAIAP2L1 while upregulating GDF15 and TP53I3/PIG3 genes predominantly associated with proliferation inhibition and cell death pathways. The heatmap in Figure 5C illustrates apoptosis-related gene expression patterns, demonstrating TP53I3/PIG3 upregulation that promoted oxidative stress-mediated apoptosis. Molecular docking analysis (Figure 5D) revealed GA's binding to MDM2 with a binding affinity of -6.91 kcal/mol. Western blot validation in Figure 5E confirmed the protein level regulation of these pathways, and in particular verified the increased expression of oxidative stress TP53I3/PIG3 and apoptosis markers.

### 4.6. Gamabufotalin Inhibited the Growth of Colorectal Cancer Xenograft Tumors in Vivo

**Figure 6.**
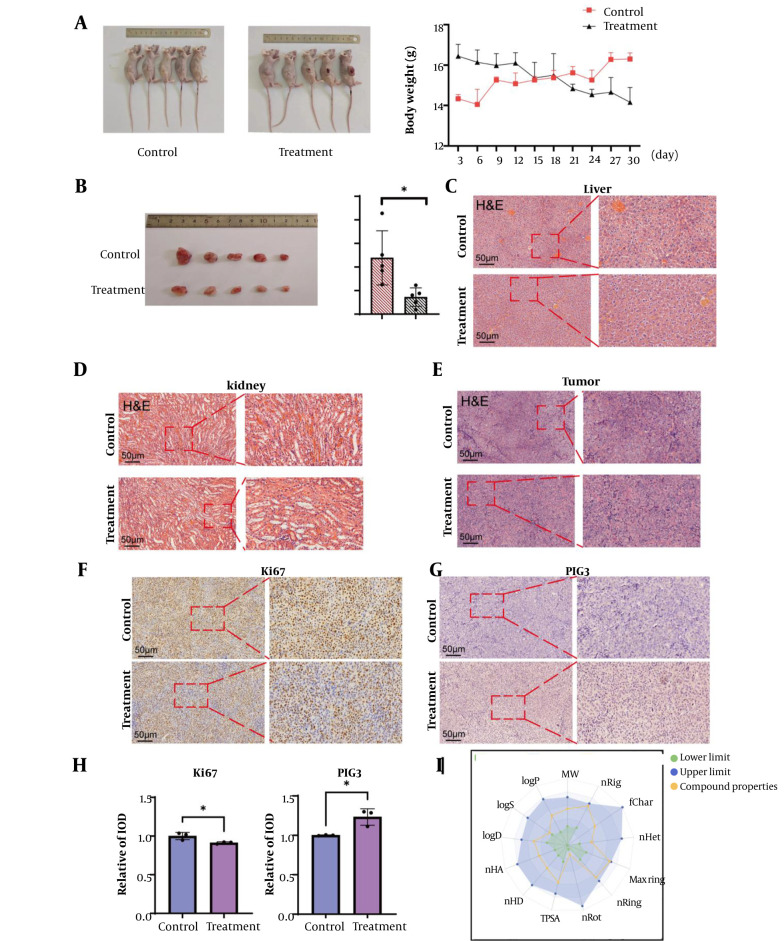
Gamabufotalin (GA) inhibits the growth of colorectal cancer (CRC) in vivo. A, after the death of the experimental mice, photos of the xenograft tumors formed in vivo were taken, and the weights of the two groups of mice were compared. B, photos of the xenograft bladder cancer tumors and the final tumor weights of the two groups were compared. Analysis was conducted using a two-tailed *t*-test, * P < 0.05. C-E, H&E staining of the liver, kidneys, and tumors of the treatment group and the control group. F-H, immunohistochemical staining was performed to detect Ki67 and the expression of the target protein TP53I3/PIG3 in the xenograft tumors. Quantification was performed using the relative optical density (IOD) method. Analysis was conducted using a two-tailed t-test, * P < 0.05. I, pharmacological analysis of GA. Data are from (https://admetlab3.scbdd.com/server/evaluationCal).

To investigate the in vivo antitumor efficacy of GA against CRC, we established subcutaneous xenograft tumor models in nude mice. Gamabufotalin treatment significantly inhibited tumor growth compared to DMSO-treated controls, as evidenced by reduced tumor volume and weight (Figure 6A and B). Histopathological evaluation through H&E staining revealed marked disruption of tumor cell architecture and decreased nuclear density in GA-treated tumors relative to controls (Figure 6C-E). Immunohistochemical analysis (Figure 6F-H) demonstrated significantly lower Ki67 expression in GA-treated groups, confirming its antiproliferative effects. Concurrently, elevated TP53I3/PIG3 expression suggested GA-induced apoptotic activity in colorectal tumor cells. Pharmacokinetic profiling of GA was additionally performed (Figure 6I). 

## 5. Discussion

Colorectal cancer remains a leading cause of death today. Targeted therapies still face challenges in terms of effectiveness and accessibility. Traditional Chinese medicine, especially the toad skin extract HCS, has shown anti-CRC potential by inducing apoptosis and improving treatment tolerance (7, 12-14). In this study, using both cell-based and animal models, we identified that GA — an active monomer found in HCS — demonstrates anti-tumor activity against CRC. The remarkably low IC_50 _value of 24 nM for GA warrants significant attention, being substantially lower than those reported for numerous conventional chemotherapeutic agents. Critically, GA's potent anti-cancer efficacy coexists with a low-toxicity profile toward normal cells. This dual characteristic — high potency against cancer cells coupled with minimal cytotoxicity in non-neoplastic cells — constitutes the cornerstone of our study's rationale. Multi-omics analyses demonstrate that GA triggers SW480 cell death by exploiting the oxidative stress-apoptosis axis. ROS critically regulate cancer cell fate through oxidative stress, influencing apoptosis, proliferation, and drug resistance. Tumor cells maintain elevated basal ROS levels due to metabolic hyperactivity, creating a fragile redox balance reliant on compensatory antioxidant defenses. This chronic oxidative stress depletes endogenous reserves, lowering their threshold for ROS tolerance (15-19). Consequently, when exogenous or endogenous factors (such as chemotherapy, radiotherapy, or targeted agents) further elevate ROS levels, they rapidly overwhelm the compensatory capacity of tumor cells. This triggers a cascade of oxidative damage — including DNA fragmentation and collapse of mitochondrial membrane potential — thereby inducing irreversible tumor cell death. This mechanism underpins therapeutic strategies targeting the oxidative stress vulnerability of tumors (17-19). Gamabufotalin demonstrated a pronounced inhibitory effect on cell viability and survival by upregulating intracellular ROS levels. Following the addition of the antioxidant NAC, there was a significant rebound in cell viability, indicating that the cells were indeed experiencing oxidative stress. P53-inducible gene 3 (TP53I3/PIG3) was initially identified through an analysis of p53-inducible genes associated with apoptosis initiation (20). TP53I3/PIG3 has been reported to possess enzymatic activity characteristic of oxyreductases, providing direct evidence for its involvement in ROS generation within cells (21). Previous studies have indicated that TP53I3/PIG3 contributes to the production of ROS, which serve as important downstream mediators in the apoptotic response (21). Furthermore, ROS are also recognized as downstream mediators of p53-dependent apoptosis (22, 23). Furthermore, the upregulation of TP53I3/PIG3 has been shown to inhibit tumor cell proliferation and induce apoptosis, indicating that TP53I3/PIG3 may serve as a potential therapeutic target for gastric cancer (20). For example, in breast cancer, BRCA1 modulates PIG3-mediated apoptosis in a p53-dependent manner (24). High-throughput proteomics and Western blot analyses revealed that GA significantly increased the expression levels of TP53I3/PIG3 and C-caspase-3. These findings suggest that GA activates the P53 signaling pathway to trigger apoptotic processes. Notably, MDM2 — an interacting protein of GA — was investigated through computational simulations. The inhibition of MDM2 leads to the activation of P53 (25, 26); activated p53 elevates ROS via upregulation of TP53I3/PIG3, initiating mitochondrial apoptosis through cytochrome c release and caspase-9/3 activation (17, 22, 27). However, several important limitations of this study should be acknowledged. First, the proposed GA-MDM2 interaction rested on computational docking rather than direct biochemical evidence. While our docking data (binding affinity: -6.91 kcal/mol) provided a plausible structural basis, and the downstream p53/PIG3 activation was functionally consistent, techniques such as surface plasmon resonance (SPR) or Biopulldown would be needed to confirm physical binding. Second, although GA consistently upregulated TP53I3/PIG3 in parallel with ROS production and apoptosis, whether PIG3 played a causal role in these effects remains unresolved. Our data established a clear association, but loss- and gain-of-function studies (e.g., PIG3 knockdown or overexpression) will be essential to determine whether this axis is indeed required for GA-induced cell death. Despite these limitations, the convergence of our multi-omics and pharmacological data strongly implicated the MDM2/p53/PIG3/ROS axis in GA's anti-CRC activity. In-depth investigation of these questions will help to fully elucidate the molecular basis and translational potential of this promising natural compound. These findings support GA's therapeutic potential, while acknowledging possible additional mechanisms. Moreover, GA also demonstrates favorable oral bioavailability and amorphous crystallization properties.

### 5.1. Conclusions

**Figure 7.**
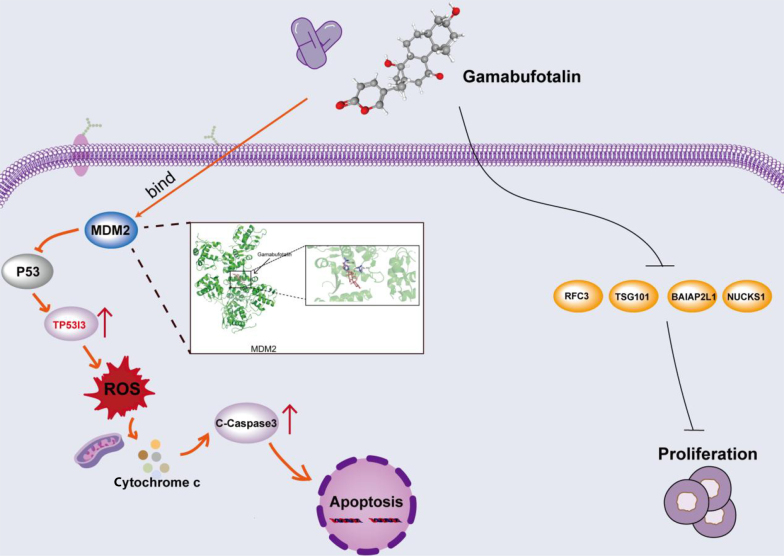
Schematic diagram of the pharmacological mechanism of gamabufotalin (GA) in combating colorectal cancer (CRC). GA primarily exerts its inhibitory effects on CRC through the induction of oxidative stress and apoptosis. The pharmacological mechanisms by which GA influences CRC are as follows: (1) GA upregulates TP53I3/PIG3, leading to an increase in ROS levels within cells, thereby triggering oxidative stress that subsequently induces apoptosis. (2) GA inhibits cell proliferation by downregulating several key proteins, including RFC3, DHFR, NUCKS1, TSG101, and BAIAP2L1. GA promotes cell death by inducing apoptosis in CRC cells while simultaneously inhibiting cell proliferation.

Collectively, GA exerts potent anti-CRC effects through dual-pronged mechanisms (Figure 7 MDM2 inhibition-triggered p53 activation upregulates TP53I3/PIG3 to amplify ROS-dependent apoptosis via caspase-9/3 cascade, and 2) synergistic suppression of oncogenic proliferators (e.g., RFC3, NUCKS1, TSG101, and BAIAP2L1). This work mechanistically validates a traditional medicine monomer as a novel redox/proliferation dual-inhibitor, providing a translational paradigm for leveraging tumor oxidative vulnerability in CRC targeted therapy.

## Data Availability

Publicly available datasets were analyzed in this study. These data can be found here: The mass spectrometry proteomics data have been deposited to the ProteomeXchange Consortium (https://proteomecentral.proteomexchange.org) via the iProX partner repository with the dataset identifier PXD065299. The transcriptome data are uploaded to the NCBI Sequence Read Archive and are available via http://www.ncbi.nlm.nih.gov/bioproject/1348147 (Bioproject ID: PRJNA1348147).
